# Basic, Translational, and Clinical Research on Dementia

**DOI:** 10.3390/ijms25136861

**Published:** 2024-06-22

**Authors:** Giacinto Bagetta, Daniele Bano, Damiana Scuteri

**Affiliations:** 1 Department of Pharmacy, Health Science and Nutrition, University of Calabria, 87036 Rende, Italy; g.bagetta@unical.it; 2 German Center for Neurodegenerative Diseases (DZNE), 53127 Bonn, Germany; daniele.bano@dzne.de; 3 Department of Health Sciences, University “Magna Graecia” of Catanzaro, 88100 Catanzaro, Italy

The global impact of dementia is an increasing area of concern and, according to the Alzheimer’s Disease International (ADI) World Alzheimer Report 2021, up to 90% of dementia patients in low- and middle-income countries are not diagnosed [1]. Approximately 57.4 million people all over the world are affected by dementia, and this number is expected to triple to 152.8 by 2050, with a female-to-male ratio of 1.67 [1]. In this regard, among the most common forms of dementia, 60–80% are caused by Alzheimer’s disease (AD) [2]. Although AD accounts for almost two-thirds of all cases, the incidence of other mixed forms of dementia (such as frontotemporal, Lewy body, etc.) is rising worldwide, making Alzheimer’s disease and related dementias (ADRD) a public health priority [3]. There is little doubt that AD is a multifactorial disease, which involves diverse pathogenic mechanisms and will probably require combinatorial therapies. Although many pathological hallmarks have been widely described, such as accumulation of amyloid-beta (Aβ) aggregates and tau-containing neurofibrillary tangles, the mechanism underlying proteotoxicity and its role in combination with other factors at different disease stages [4,5] requires further clarification. In fact, much more research is needed regarding a better definition of the dementia pathophysiology and to better understand the mechanism of action of natural products in disorders of neuronal pathways [6,7].

Among several markers, the nanosized extracellular vesicles (EVs), representing important mediators of cellular communication, are promising candidate biomarkers for neurodegenerative diseases like AD. Visconte et al. [8] have isolated total EVs from the plasma of AD patients using ExoQuickULTRA exosome precipitation solution (SBI). Circulating total EVs were characterized using Nanosight nanoparticle tracking analysis (NTA), transmission electron microscopy (TEM), and Western blotting. A panel of 754 miRNAs was determined with RT-qPCR using TaqMan OpenArray technology in a QuantStudio 12K System (Thermo Fisher Scientific, Waltham, Massachusetts, United States). These novel results have demonstrated that plasma EVs show widespread deregulation of specific miRNAs, some of which are already known to be associated with neurological pathologies. A further validation analysis also confirmed significant upregulation of miR-16-5p, miR-25-3p, miR-92a-3p, and miR-451a in prodromal AD patients, suggesting these dysregulated miRNAs may be associated with the early onset of AD pathology [8].

Bonomi et al. have confirmed that the APOE ε genotype is linked with different vascular responses in AD pathology [9]. The relationship between amyloid pathology and nitric oxide (NO) dynamics has been evaluated by cerebrospinal fluid (CSF) levels measurement of neuronal Nitric Oxide Synthase (nNOS) and endothelial Nitric Oxide Synthase (eNOS) of patients with a clinical diagnosis of AD and isolated CSF amyloid changes, stratified according to APOE ε genotype (APOE ε3 = 13, APOE ε4 = 14). In this cohort, both eNOS and nNOS levels were increased in APOE ε3 with respect to healthy controls and APOE ε4. CSF eNOS was inversely correlated with CSF Amyloid-β42 selectively in carriers of APOE ε3; while CSF nNOS was negatively associated with age and CSF p-tau only in the APOE ε4 subgroup. Increased eNOS could represent compensative vasodilation to face progressive Aβ-induced vasoconstriction in APOE ε3, while nNOS could represent the activation of NO-mediated plasticity strategies in the same group [9]. This is also noteworthy in light of the full approval of lecanemab by the Food and Drug Administration (FDA) [10,11]. In fact, according to the results of the Clarity phase III clinical trial, amyloid-related imaging abnormalities (ARIA) with edema or effusions (E) and ARIA with cerebral microhemorrhages, cerebral macrohemorrhages, or superficial siderosis (H) have occurred less frequently among APOE ε4 noncarriers, with higher frequency among ApoE ε4 homozygotes [12]. Reveglia et al. [13] contributed to the debate regarding the factors that trigger AD by means of their metabolomic data, confirming that AD is more than a brain disease and harms the whole-body metabolism. In fact, multivariate statistical analysis showed that there were at least 25 significantly dysregulated metabolites from plasma of patients with AD (n = 20) compared with the healthy controls (n = 20). Two membrane lipid components, glycerophospholipids and ceramide, were upregulated, whereas glutamic acid, other phospholipids, and sphingolipids were downregulated. These results demonstrated that at least five pathways involved in the metabolism of polar compounds undergo dysregulation in patients with AD; conversely, the lipid pathways have not shown significant alterations. These results support metabolome analyses to understand alterations in the metabolic pathways related to AD pathophysiology [13].

On the other hand, inflammatory mechanisms are increasingly recognized as important contributors to the pathogenesis of neurodegenerative diseases. Autoantibodies directed against N-methyl-D-aspartate (NMDA) and α-amino-3-hydroxy-5-methyl-4-isoxazolepropionic acid (AMPA) receptors have been reported in the central nervous systems of patients suffering from brain disorders characterized by neurological and psychiatric symptoms. Their impact on the functions and/or the expression of the targeted receptors can alter synaptic communication. Olivero et al. [14] have attempted to clarify the molecular mechanisms involved in the autoantibody-mediated effects in preclinical models, in order to understand their pathogenic role in central disorders, but also to propose new therapeutic approaches for preventing the deleterious central consequences. NMDA receptors are tetramerically assembled, including the obligatory GluN1 subunits associated with the GluN2 (A-D) and/or the GluN3 (A-B) subunits [15]. The results obtained with the anti-GluA autoantibodies are highly heterogenous and more investigations are needed to clarify their role in dictating the changes in AMPA receptor expression and functions. On the contrary, the results concerned with anti-GluN antibodies are consistent with an antibody-induced internalization of the NMDA receptor subunit that well accounts for the synaptic desynchronization that supports some of the clinical manifestations observed in patients [14].

Inflammatory or exogenous molecules leading to sustained neuroinflammation are the consequences of altered metabolic status of individuals with nonalcoholic fatty liver disease (NAFLD). The latter molecules can directly affect brain activity, primarily by altering blood–brain barrier (BBB) integrity, thus allowing their sustained transit, leading to impaired cognition or dementia. This is the view discussed by Giuffrè et al. [16], also supporting gut dysbiosis as a crucial role by altering the gut barrier and its interplay with the liver, allowing the entrance of bacterial products that can promote systemic inflammation. Moreover, several factors, including dietary vitamin deficiencies, can promote Aβ-plaques deposition, thus worsening AD outcomes [17].

Through the scoping approach [18], Loveland et al. [19] have examined methods for the investigation of inflammation in dementia with Lewy bodies (LBD) and Parkinson’s disease dementia and have identified alterations in inflammatory signals in LBD compared to people without neurodegenerative disease and other neurodegenerative diseases. The results of their systematic scoping review point at innate and adaptive immune system contributions to inflammation associated with Lewy body pathology and clinical disease features. Also, different signals in early- and late-stage disease, with possible late immune senescence and dystrophic glial cell populations, have been identified, though the limited strength of these associations is due to varying methodologies, small study sizes, and the cross-sectional nature of the data, suggesting that longitudinal studies investigating associations with clinical and other biomarker outcomes are needed [19].

Ribeiro et al. [20] have reported on the difficulties of assessing and managing pain, under conditions when obtaining a self-report is impossible and therapeutic decision-making becomes more challenging. On the basis of their experimental data, they proposed monocytes and some membrane monocyte proteins, identified as a cluster of differentiation (CD), as potential, non-invasive, peripheral biomarkers in identifying and characterizing pain in patients with severe dementia. Their preliminary data indicate that the relative concentrations of monocytes, particularly the percentage of classic monocytes, may be a helpful pain biomarker. Indeed, recognizing pain in patients who are unable to communicate is of great significance. Hence, objective biomarkers along with appropriate observational clinimetric tools [21] would represent an approach worthy of investigation. Moreover, pain responses can provide valuable insights into a patient’s condition. In patients with disorders of consciousness (DOC), the presence of pain might indicate residual or covert consciousness, influencing prognostic considerations and care plans. In DOC patients, difficulties in accurately predicting an individual’s ability to experience pain and distress are very difficult [22]. An accurate assessment of pain responses can provide insights into changes in the individual’s level of consciousness, further emphasizing the ongoing importance of managing pain in these patients, as highlighted by Riganello et al. [23].

Another aspect of great importance is obtaining a better understanding of the complex disease aetiology, including the identification of early disease markers aided by ultrastructural magnetic resonance imaging to develop more effective treatments. Martucci A et al. [24] have pointed out the overlapping of magnetic resonance imaging (MRI) and clinical biomarkers between glaucoma and AD, summarizing the current state of the art on the use of advanced neuroimaging techniques in neurodegenerative diseases. Glaucoma is a multifactorial degenerative optic neuropathy, representing the second leading cause of blindness worldwide [25,26]. It is classically associated with structural and functional changes in the optic nerve head and retinal nerve fiber layer [27], but the damage is not limited to the eye, leading to the use of neuroprotective agents without conclusive evidence [28]. Involvement of the central visual pathways and disruption of brain network organization have been reported using advanced neuroimaging techniques [29]. The brain structural changes at the level of the areas implied in processing visual information could justify the discrepancy between signs and symptoms and underlie the analogy of this disease with neurodegenerative dementias, such as AD, and with the complex group of pathologies commonly referred to as “disconnection syndromes” [24,30].

AD and epilepsy are common neurological disorders in the elderly, with epilepsy being the third most common neurological disorder affecting them after stroke and dementia [31]. Thus, the relationship between epilepsy and AD represents a challenge as well as a continuing need. Seizures in AD patients are often unrecognized because they are often nonconvulsive and sometimes mimic some behavioral symptoms [32]. Interestingly, preclinical studies have shown that some antiseizure medications (ASMs) targeting abnormal network hyperexcitability might change the natural progression of AD [33], though this needs to be demonstrated. The study by Bosco et al. [34] highlighted the need for future studies to be directed toward detecting AD patients with subclinical epileptiform activity and to definitely establish the usefulness of ASMs in ADRD.

Drug repurposing is generating a wide number of clinical studies, although they are often disappointing. One of the reasons is the lack of proper candidate selection. More in-depth analysis of the literature may offer opportunities to build a stronger rationale to predict successful clinical trials. Consistently, several drugs belonging to different classes have been suggested to be effective in managing AD by means of autophagy induction [35,36,37]. Useful autophagy inducers in AD should be endowed with a direct, measurable effect on autophagy, have a safe tolerability profile, and have the capability to cross the BBB, at least with poor penetration. According to the PRISMA 2020 recommendations, Corasaniti et al. [38] have conducted a systematic review to appraise the measurable effectiveness of autophagy inducers in the improvement of cognitive decline and neuropsychiatric symptoms in clinical trials and retrospective studies. The outcomes most influenced by the treatment were cognition and executive functioning, pointing at a role for metformin, resveratrol, masitinib and TPI-287, with an overall tolerable safety profile. Differences in sample power, intervention, patients enrolled, assessment, and measure of outcomes prevent the generalization of results. Moreover, the domain of behavioral or neuropsychiatric symptoms has been found to be less investigated, thus prompting new prospective studies with homogeneous design [38] since the latter affect some 99% of patients over the course of the disease [39] and they are managed with potentially harmful antipsychotics [40]. Belardo et al. [41] have attempted a preclinical approach to the development of novel non-pharmacological control of AD and associated neuropsychiatric symptoms. They investigated transient and persistent global amnesia which is a very common neuropsychiatric syndrome. Among animal models for amnesia and for testing new drugs, the scopolamine test is the most widely used for transient global amnesia (TGA) [42]. In C57BL/6 mice treated with intraperitoneal scopolamine (1 mg/kg), the authors investigated the effects of intranasal palmitoylethanolamide, 2-pentadecyl-2-oxazoline (PEA-OXA; 10 mg/kg). Scopolamine induced deficits of discriminative and spatial memory and motor deficit. These changes were associated with a loss of synaptic plasticity in the hippocampal dentate gyrus and impaired long-term potentiation (LTP) after lateral entorhinal cortex/perforant pathway tetanization. Furthermore, hippocampal levels of acetylcholine decreased. PEA-OXA has either prevented or restored the scopolamine-induced cognitive deficits (discriminative and spatial memory). However, the same treatment has not affected the altered motor activity or anxiety-like behavior induced by scopolamine. Consistently, electrophysiological analysis has shown LTP recovery in the dentate gyrus of the hippocampus. Therefore, this study has confirmed the neuroprotective and pro-cognitive activity of PEA-OXA (probably through an increase in the extracellular levels of biogenic amines) in improving transient memory disorders, for which the available pharmacological tools are obsolete or inadequate and are not directed on specific pathophysiological targets [41].

Nowadays, the combination of pharmacological and non-pharmacological therapies seems to be the best to stimulate cognitive reserve. Over the last twenty years, several drugs have been discovered based on the well-established biological hallmarks of AD; i.e., deposition of Aβ aggregates and accumulation of hyperphosphorylated tau. A new era in treating AD has recently emerged. Ongoing clinical trials with disease-modifying therapies (DMTs) other than lecanemab and non-pharmacological therapies are actually populating the pipeline. Buccellato et al. [43] have examined open questions arising from current clinical trials, underlining that the development of future scenarios is now possible, on the basis that new biomarkers can detect AD in preclinical or prodromal stages, identifying people at risk of developing AD, and allowing early and curative treatment. In particular, a comprehensive understanding of the complex disease mechanisms together with the identification of early disease markers aided by ultrastructural imaging is necessary to develop more effective treatments. Another fundamental aspect is represented by the gathering of information from real-world data on novel DMTs. These data may prompt the further development of clinical trials in AD.
